# MiR-206 improves intervertebral disk degeneration by targeting GJA1

**DOI:** 10.1186/s13018-022-03044-1

**Published:** 2022-03-12

**Authors:** Peng Zhou, Peng Xu, Wantao Yu, Huan Li

**Affiliations:** Department of Orthopedics and Joints, The Third Affiliated Hospital of Soochow University, No. 185, Juqian Street, Tianning District, Changzhou City, 213000 Jiangsu Province China

**Keywords:** Intervertebral disk degeneration, Nucleus pulposus, miR-206, GJA1

## Abstract

**Background:**

A large amount of evidence suggested that miRNA was involved in the progression of intervertebral disk degeneration (IDD). The purpose of our study was to explore the function and potential mechanism of miR-206/GJA1 axis in IDD.

**Methods:**

IDD nucleus pulposus (NP) cell model was established through treatment of LPS. IDD rat model was established by annulus fibrosus puncture. The expression of miR-206 and GJA1 was detected by RT-PCR, apoptosis was evaluated by flow cytometry or TUNEL, inflammatory factors were tested by ELISA, extracellular matrix related protein expression was detected by western blot, and HE and safranin-O staining were used to assess the pathological changes of IDD.

**Results:**

GJA1 was found to be highly expressed in IDD tissues and LPS-induced NP cells. Down regulation of GJA1 reduced inflammatory factors, inhibited apoptosis and enhanced extracellular matrix in LPS-induced NP cells. MiR-206 was downregulated in IDD tissues and directly targeted GJA1, and the expression of miR-206 was negatively correlated with the expression of GJA1 in IDD tissues. Further, it was demonstrated that overexpression of miR-206 could attenuate LPS-induced NP cell injury by targeting GJA1. In vivo, the upregulation of miR-206 improved IDD and reduced NP cell apoptosis.

**Conclusion:**

Our study showed that miR-206 reduced the level of inflammatory factors, restrained NP cell apoptosis and increases extracellular matrix by targeting GJA1. These data suggested that miR-206/GJA1 might be potential therapeutic targets for IDD.

## Introduction

Low back pain seriously affects people's life. Intervertebral disk degeneration (IDD) is one of the main causes of low back pain [1–3]. IDD is commonly characterized by increased fibrosis of nucleus pulposus (NP), damage of fibrous ring and calcification of cartilage endplate, which usually lead to lumbar disk herniation, lumbar spinal stenosis, lumbar spondylolisthesis, lumbar instability, etc. [4–6]. These diseases clinically show radiation pain in the back and lower limbs. Moreover, IDD becomes more and more serious with the increase of population age [7, 8]. IDD not only brings pain to patients, but also enhances economic burden to families and society. The pathogeny of IDD is very complex, and the traditional view is that age, gender, height, smoking, weight bearing and physical exercise accelerate intervertebral disk degeneration [9, 10]. However, with the researchers' in-depth understanding of the mechanism of IDD at the cellular and molecular level, the mechanism of IDD is related to the increase of inflammatory factors and degradation of extracellular matrix (ECM), and the apoptosis of NP cells [11–13]. Therefore, the study of the molecular mechanism is of great significance to clarify the pathogenesis and treatment of IDD.

MicroRNA (miRNA) is an endogenous and single-stranded small RNA composed of 18–25 nucleotides and could not encode protein [14]. MiRNAs are widely involved in the post transcriptional regulation of protein expression and plays a key regulatory role in the stability of mRNA [15, 16]. More and more evidences show that miRNA is closely related to the regulation of many biological progression [17–19]. At present, exploring the miRNA involved in IDD has become a research hotspot [20, 21]. The study has shown that miR-7 is highly expressed in Interleukin-1β (IL-1β) induced NP cells, and miR-7 silencing could inhibit IL-1β induced NP cell injury by targeting growth differentiation factor 5 [22]. It was found that miR-155 overexpression could slow down IDD through upregulating MMP-16 [23]. MiR-206 is involved in regulating the physiological progression of many diseases including cancer, osteoarthritis, osteoporosis, ulcerative colitis and so on [24–27]. However, the role and mechanism of miR-206 in IDD are still unclear.

Through Gene Expression Omnibus (GEO) database analysis, we found that GJA1 was overexpressed in IDD tissues. Moreover, only miR-206 was lowly expressed in IDD tissues among target miRNA of GJA1. Further functional experiments indicated that miR-206 inhibited NP cell apoptosis, inflammation, amplified ECM by targeting GJA1, and the overexpression of miR-206 could improve rat IDD in vivo.

## Materials and methods

### Database analysis

GSE34095 dataset was downloaded from GEO database, including three IDD tissues and three normal intervertebral disk tissues. Differential expression genes were analyzed according to the GPL96 platform (Affymetrix human genome U133A array). The up-regulated and down-regulated mRNA of top 20 were shown by heat map. MiRNAs targeting the GJA1 gene were analyzed by targetscan_7.1 (TargetScanHuman 7.1).

### Tissue samples

20 IDD tissues and 20 normal intervertebral disk tissues were, respectively, collected form 20 patients with intervertebral disk degeneration and 20 patients with traumatic lumbar fracture The patients were treated in the Third Affiliated Hospital of Soochow University hospital from March 2019 to September 2020. IDD was evaluated according to spinal surgical pathology. The study was approved by ethics association of the Third Affiliated Hospital of Soochow University hospital. All participants have signed the informed consent form.

### Isolation of NP cell

NP tissue was isolated from the normal intervertebral disk tissues. In sterile environment, the NP tissue was washed with D-hanks buffer for five times, cut into small pieces with sterile surgical scissors, and then digested for 4 h at 37 °C using 0.25% trypsin. NP cells were collected by centrifugation (10 min, 1000 rpm), resuspended with DMEM medium containing 10% fetal bovine serum, and then cultured in 37 °C, 5% CO_2_ incubator. The third generation NP cells were used in subsequent experiments.

### Cell transfection

MiR-206 mimics, mimics NC, miR-206 inhibitor, inhibitor NC, sh-GJA1, sh-NC, pcDNA-GJA1 and vectors were synthesized and purchased from GenePhama Biotechnology (Shanghai). These RNAs were transfected into NP cells using Lipofectamine 2000 (Thermo Fisher) according to the application instructions. For the experiments involved in lipopolysaccharide (LPS) treatment, after transfection of 24 h, NP cells were treated for 24 h with 100 ng LPS, and the same amount of normal saline was used as the control, and then the subsequent experiments were performed.

### Reverse transcription-polymerase chain reaction (RT-PCR)

Total RNA in NP cells was isolated by Trizol (Invitrogen). cDNA mRNA and miRNA were, respectively, synthesized using iScript Select cDNA Synthesis kit (BIO-RAD) and TaqMan® MicroRNA Reverse Transcription Kit (Invitrogen). RT-PCR of mRNA and miRNA were, respectively, performed with SYBR Green realtime PCR Master Mix (Toyobo) and TaqMan microRNA assay kit (Applied Biosystems, USA) on an ABI 7500 System. The reaction conditions were as follow: pre-denaturation at 95 °C for 3 min, and then 40 cycles (denaturation at 95 °C for 15 s, annealing at 60 °C for 20 s, extension at 72 °C for 20 s). U6 or GAPDH are used as internal parameters. The data were analyzed according to 2^−△△Ct^ method.

### Dual luciferase reporter assay

The sequence based on wild-type 3' UTR region or the mutated 3' UTR region of GJA1 combined with miR-206 was designed and constructed by GenePharma Co., Ltd. (Shanghai, China), and which was inserted into pmirGLO plasma (Promega, Madison, WI, USA) to obtain the luciferase reporter vectors, and named as GJA1-WT or GJA1-MUT, respectively. The mimics NC and miR-206 mimics were transfected into NP cells using Lipofectamine 2000 (Thermo Fisher). After transfection of 48 h, the fluorescence intensity of luciferase was evaluated by the Luciferase Reporter Assay System (Promega).

### Cell apoptosis assay

After cultured for 48 h, NP cells were collected. Following that 5 μL Annexin V/FITC (15 min) and 10 μL propidium iodide solution (5 min) were used for staining the cells at room temperature in dark, respectively. Then, flow cytometry (BD Biosciences) was used to measure cell apoptosis, and the results were analyzed by CellQuest Pro software (BD Biosciences). The experiment was replicated three times independently.

### Western blot

The total protein was extracted using RIPA reagent (Beyotime), and the protein concentration was detected using BCA detection kit (Beyotime). 40 μg protein sample/lane was subjected to 10% SDS-PAGE and then transferred to PVDF membranes. The membranes were sealed with 5% skimmed milk at room temperature and then incubated at 4℃ overnight with the corresponding primary antibody. Following that the membranes were incubated for 2 h at room temperature with the corresponding secondary antibody. Protein bands were developed using ECL (Millipore), and images were taken and analyzed using Bio-Rad ChemiDoc XRS + (Bio-Rad, CA, USA). Primary antibodies (Anti-GJA1 (1:1000), anti-Collagen II (1:1000), anti-MMP-3 (1:2000), anti-MMP13 (1:2000), anti-ADAMTS4 (1:1000), anti-ADAMTS5 (1:1000), anti-GAPDH (1:5000)) and HRP labeled secondary antibodies (1:5000) were obtained from Abcam (Cambridge, USA). GAPDH was the normalization.

### ELISA

After adjusting the cell concentration to 2 × 10^6^/ml, the cells were inoculated into six well plates and cultured at 37 °C for 24 h in a 5% CO_2_ incubator. The cells were treated according to the group. Following that, the cell supernatant was collected by centrifuging at 3000 rpm for 10 min, and then the contents of IL-1β, TNF-α and IL-6 were measured according to the instructions of the corresponding kits. The experiment was repeated at least three times.

### Rat model of IDD

All animal experiments were performed in accordance with the Animal Care and Use Committee of the Third Affiliated Hospital of Soochow University. 24 Sprague–Dawley (SD) rats were randomly divided into four groups: sham group (Sham, n = 6), model group (Model, n = 6), agomiR-NC group (AgomiR-NC, n = 6) and agomiR-206 group (AgomiR-206, n = 6). The rats were anesthetized by intraperitoneal injection of 90 mg/kg ketamine and 10 mg/kg xylazine, and the operation was performed until the desired anesthetic effect was achieved. The rats were fixed on the operating table in prone position. In the sham operation group, only skin incision and suture were performed along spinous process of back. In the model, agomiR-NC, and agomiR-206 group, the posterior median incision was performed along spinous process of back, the exposed L3/4 intervertebral disk was punctured into 3.0 mm using a 21-gauge needle, and the needle was rotated 360° along the axis twice and maintained for 30 s. These accelerated degeneration of intervertebral disk. Following that the wound was sutured, and the rats were treated with intramuscular injection of penicillin to resist infection.

### H&E and Safranin-O staining

The NP tissue was fixed with formalin for 24 h, and then routine paraffin Sects. (4.5 μm) were performed. The slices were placed in the oven (70 °C) for 1 h, immersed in xylene for 30 min, rehydrated with gradient concentration of absolute ethanol (100%, 95%, 85%, 75%), stained with hematoxylin for 3 min, and then treated with 2% acetic acid for 1 min and ammonia for 1 min. For H&E staining, the sections were treated for 5 s with 95% absolute ethanol, and then stained for 2 min with eosin staining solution. For safranin-O staining, the sections were stained with fast green for 6 min, differentiated with 1% acetic acid for 15 s, and then stained with safranin-O for 6 min. Subsequently, the slices were dehydrated using gradient concentrations of absolute ethanol (75%, 85%, 95%, 100%), treated for 6 min with xylene solution, and then sealed with neutral resin. The results of six random fields were observed and photographed in an optical microscope (Olympus, IX‐71).

### TUNEL assay

The NP tissue sections were stained according to the instructions of TUNEL kit (Roche, Switzerland), DAPI (Beyotime) was used to stain the nucleus. The apoptosis of cells in five random fields of NP was observed under a fluorescence microscope (Olympus, BX53). The nuclei labeled with red fluorescence were TUNEL positive cells. The results were analyzed using image pro plus 6 software.

### Statistical analysis

Prism GraphPad 6.0 (San Diego, USA) was used to analyze our data. Quantitative data were presented as means ± SEM. The comparisons were performed via Student’s t-test or ANOVA (one-way). *P* < 0.05 was represented statistically significant.

## Results

### GJA1 was upregulated in IDD tissues

**Fig. 1:**
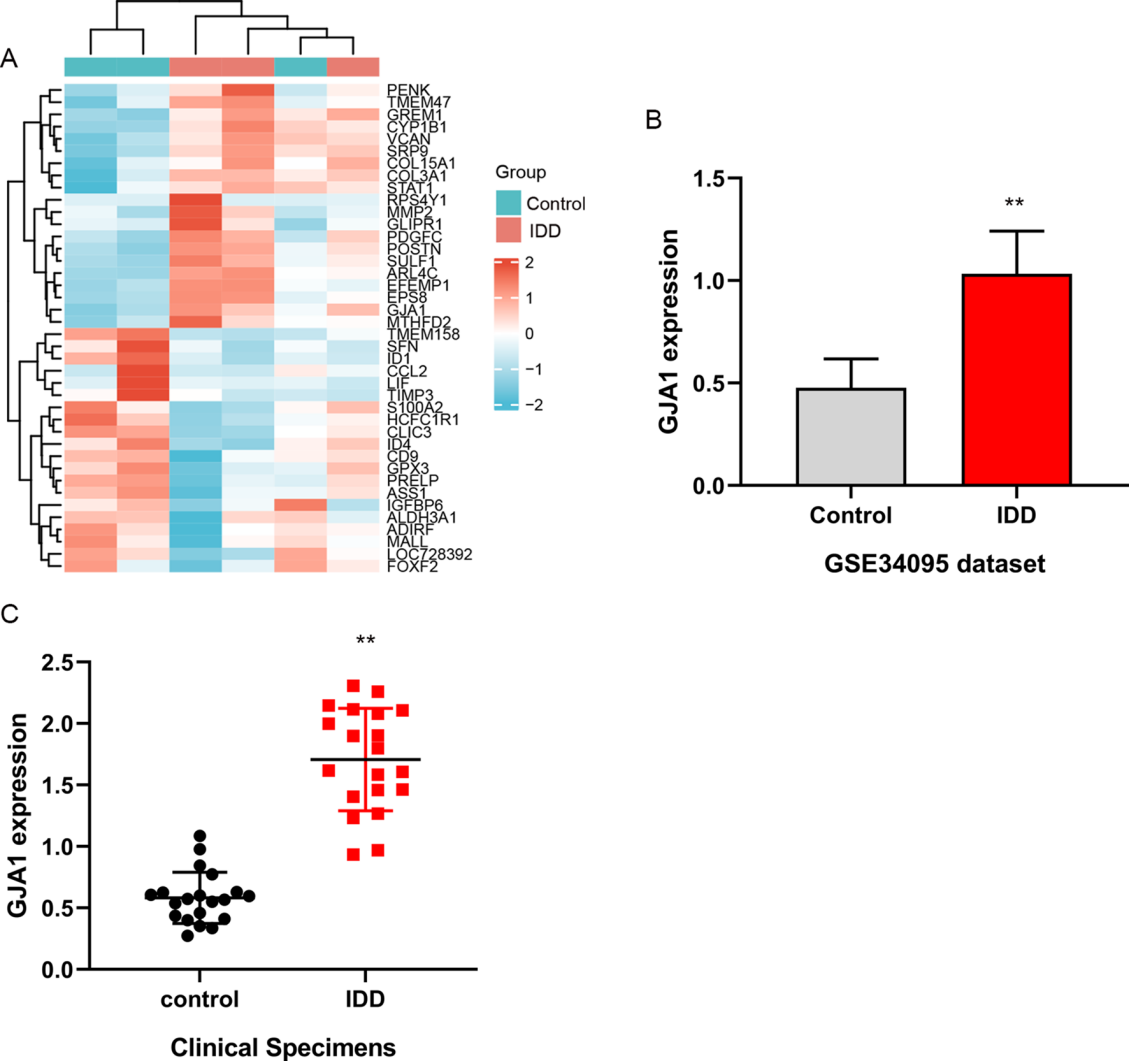
GJA1 was overexpressed in IDD. **A** Top 20 upregulated and downregulated differential expression genes between IDD tissues and normal intervertebral disk tissues in GSE34095 dataset. **B** GJA1 mRNA level of IDD tissues and normal intervertebral disk tissues in GSE34095 dataset. **C** The expression of GJA1 mRNA was detected by RT-PCR in clinical IDD tissues and normal intervertebral disk tissues. **P < 0.01 versus control group

Based on the results of analyzing GSE34095 dataset, the GJA1 in up-regulated mRNA of top 20 is highly expressed in IDD tissues compared with normal intervertebral disk tissues (Fig. 1A, B). Moreover, the function of GJA1 has not been studied in IDD, which was selected as our research object. Similarly, the results of RT-PCR showed that GJA1 was overexpression in IDD tissues than that in normal intervertebral disk tissues (Fig. 1C). These results showed that GJA1 was abnormally upregulated in IDD tissues.

### Silencing GJA1 inhibit the inflammation and apoptosis and enhance the extracellular matrix in NP cells induced by LPS

**Fig. 2:**
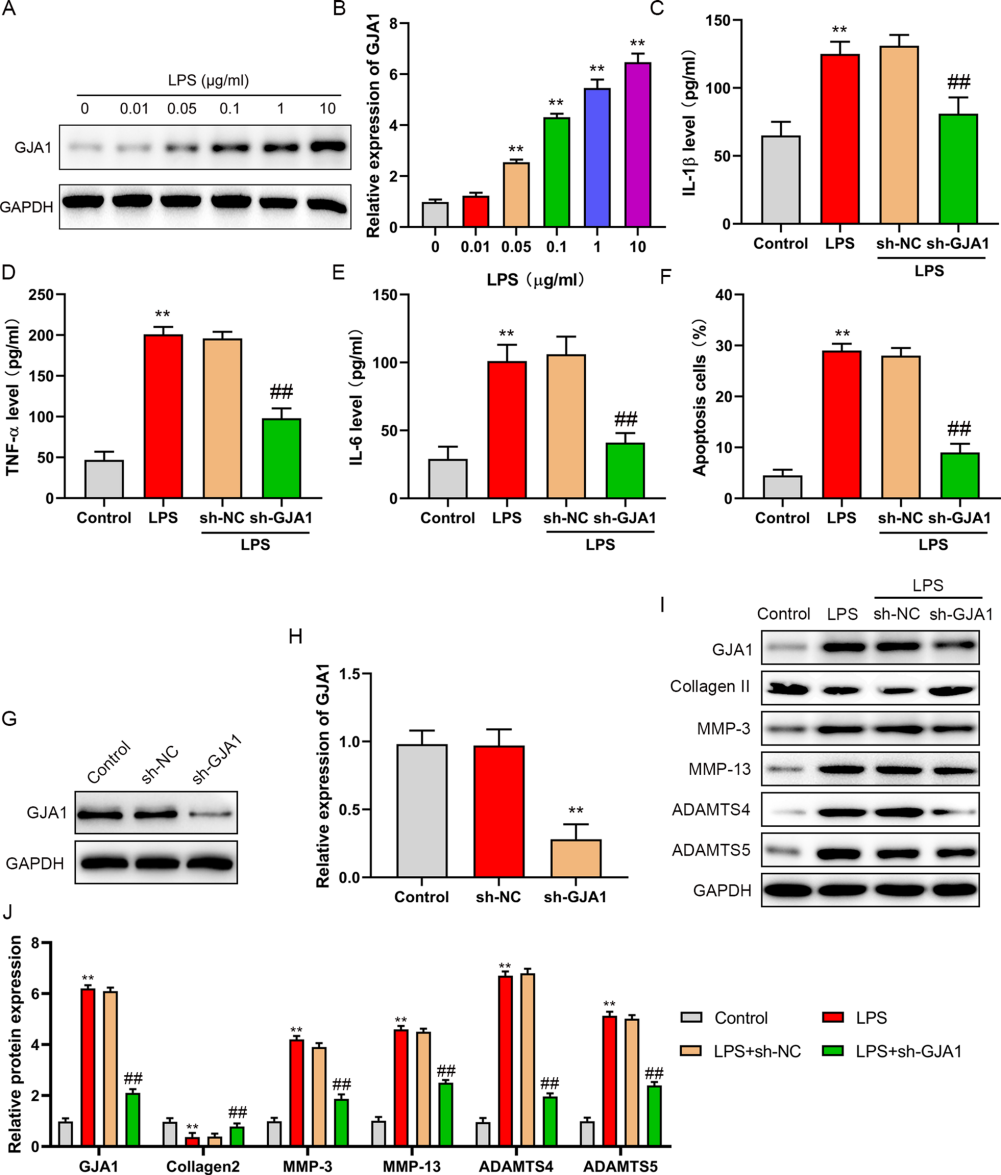
GJA1 increased inflammation, promote apoptosis, decrease extracellular matrix (ECM) in LPS-induced nucleus pulposus (NP) cells. **A**, **B** NP cells were treated with LPS (0, 0.01, 0.05, 0.1, 1, 10 μg/ml), and then GJA1 expression was measured by western blot. **C**–**E** NP cells were treated with PBS, LPS, LPS + sh-NC, or LPS + sh-GJA1, the levels of IL-1β, TNF-α, and IL-6 were detected by ELISA. **F** NP cells apoptosis was detected by flow cytometry. **G**, **H** NP cells were transfected with GJA1, GJA1 expression was tested by western blot. **I**, **J** GJA1, Collagen II, MMP-3, MMP-13, ADAMTS4 and ADAMTS5 were evaluated by western blot. **P < 0.01 versus 0 μg/ml LPS group, **P < 0.01 versus control group, ##*P* < 0.01 versus sh-NC group

An IDD cell model was established by treating NP cells with LPS. NP cells were treated with different concentrations of LPS for 24 h, the expression of GJA1 was enhanced with the increase of LPS concentration (Fig. 2A, B). GJA1 was downregulated in NP cells transfected with sh-GJA1 (Fig. 2G, H). Further, our finding indicated that the downregulation of GJA1 could reduce the increase of inflammatory factors (IL-1β, TNF-α and IL-6) and inhibit apoptosis in NP cells induced by LPS (Fig. 2C–F). In addition, the decrease of type II collagen, the increase of MMP-3, 13 and ADAMTS4 and 5 were reversed by sh-GJA1 transfection in NP cells induced by LPS (Fig. 2I, J). These results suggested that blocking GJA1 could reduce inflammatory, inhibit apoptosis and amplify ECM in LPS-induced NP cells.

### MiR-206 targets GJA1 in NP cells

**Fig. 3:**
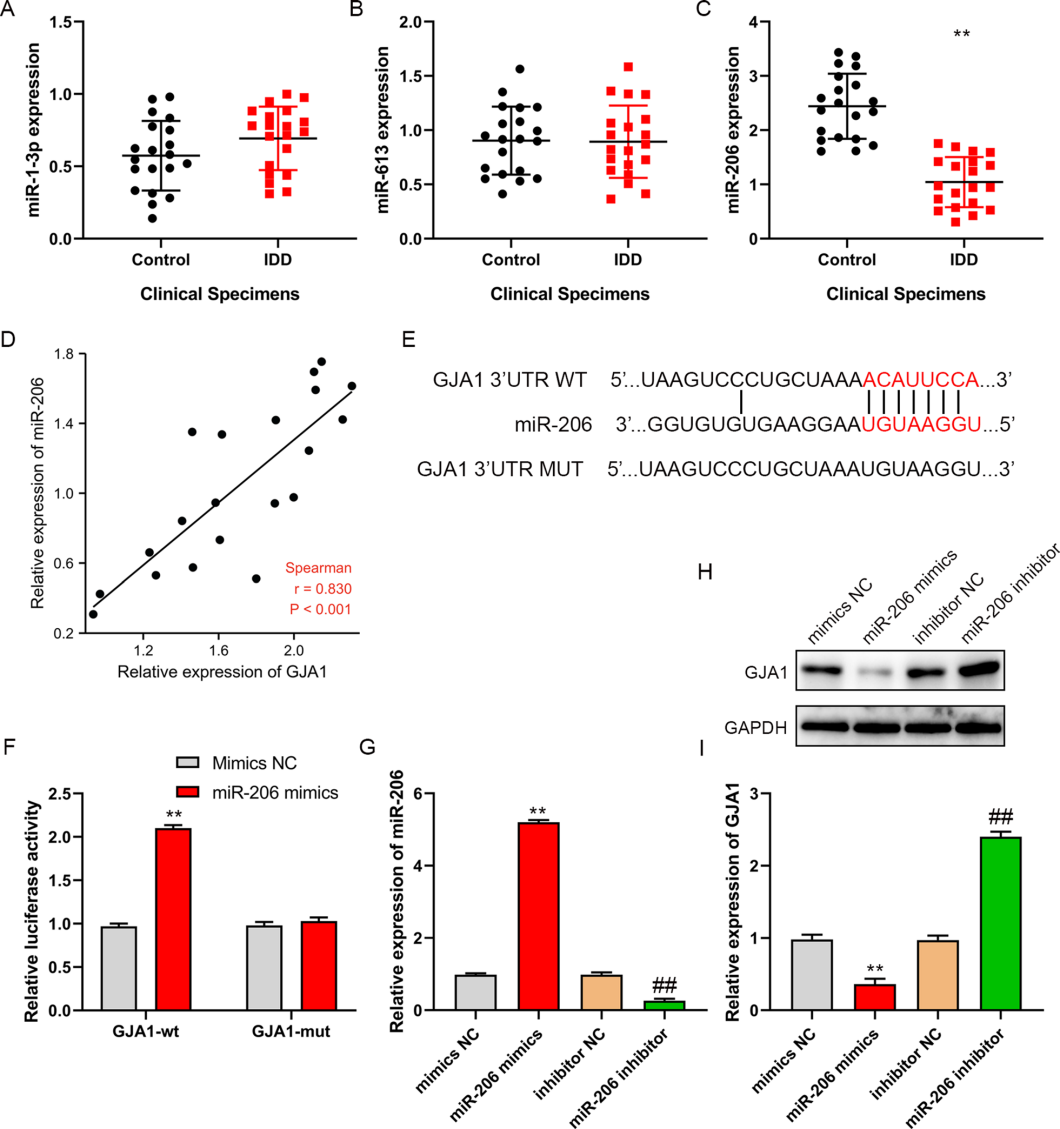
miR-206 targeted GJA1. **A**–**C** miR-1-3p, miR-613 and miR-206 expression were detected by RT-PCR in clinical IDD tissues and normal intervertebral disk tissues. **D** Correlation analysis was performed between miR-206 and GJA1 in IDD tissues. **E** Binding sites of miR-206 and GJA1 were predicted through Targetscan_7.1 (TargetScanHuman 7.1). **F** The relationship between miR-206 and GJA1 was verified by luciferase reporter gene. **G** NP cells were transfected with inhibitor NC, miR-206 inhibitor, mimics NC, or miR-206 mimics, the expression of miR-206 was detected by RT-PCR. **I** GJA1 expression was measured by western blot. **P < 0.01 versus control group, **P < 0.01 versus mimics NC group, ##*P* < 0.01 versus inhibitor NC group

Further, it was found that miRNAs targeting GJA1 included miR-1-3p, miR-206 and miR-613 through Targetscan. Moreover, miR-206 was downregulated in IDD tissues, but there was no significant difference in the expression of miR-1-3p and miR-613 between IDD tissues and normal intervertebral disk tissues (Fig. 3A–C). Besides, miR-206 expression was negatively correlated with the expression of GJA1 in IDD tissues (Fig. 3D). In addition, binding sites of miR-206 and GJA1 were analyzed through Targetscan (Fig. 3E), and luciferase reporter gene assay verified that miR-206 directly targeted GJA1 (Fig. 3F). MiR-206 mimics could increase miR-206 and inhibit GJA1 expression, on the contrary, miR-206 inhibitor could decrease miR-206 and enhance GJA1 expression (Fig. 3G–I). These demonstrated that miR-206 directly targets GJA1 in NP cells.

### MiR-206 restrains inflammation and apoptosis of NP cells, enhances ECM by targeting GJA1

**Fig. 4:**
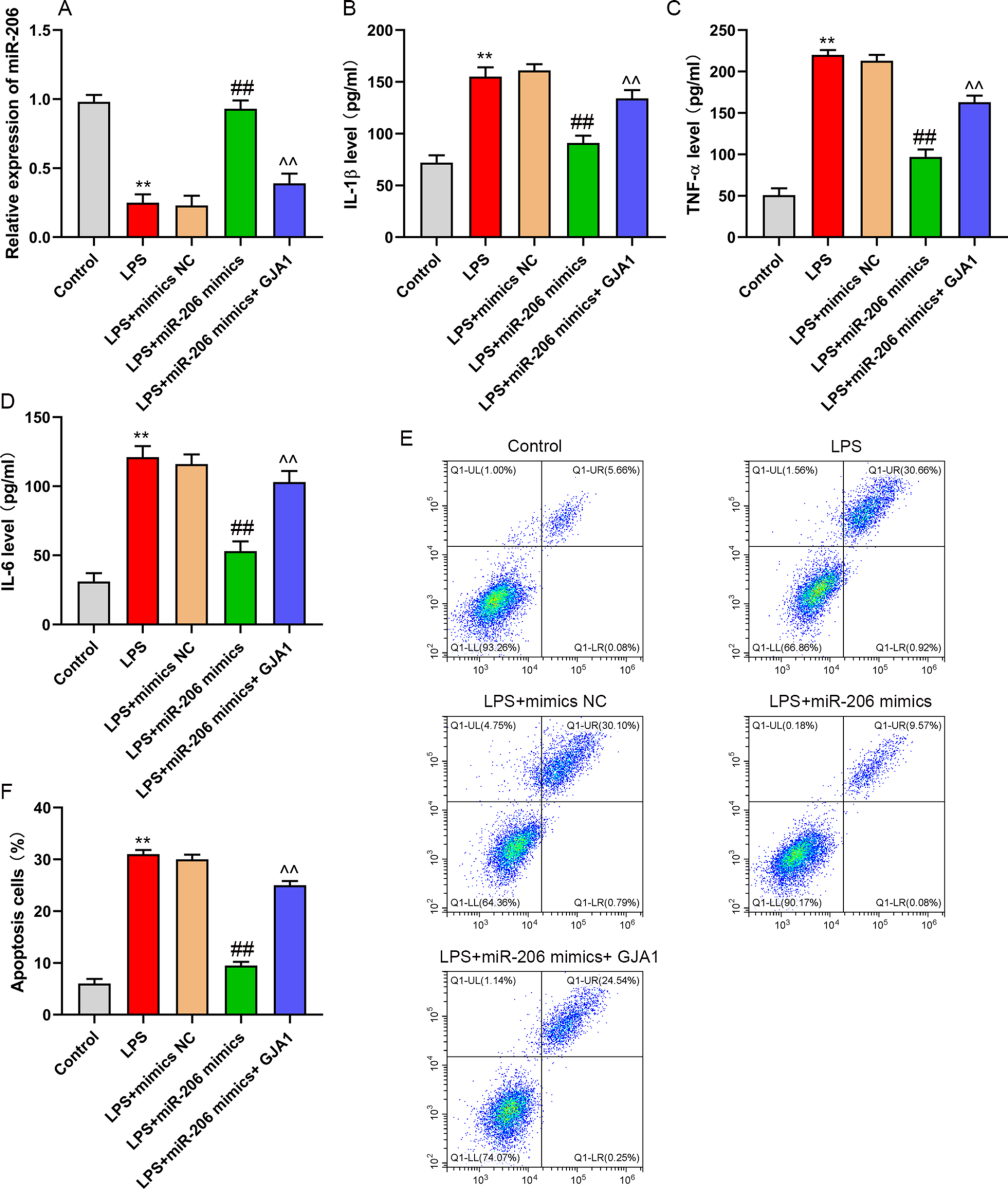
upregulation of miR-206 reduced inflammation and inhibited apoptosis by targeting GJA1 in LPS-induced NP cells. NP cells were treated with PBS, LPS, LPS + mimics NC, LPS + miR-206 mimics, or LPS + miR-206 mimics + GJA1. **A** miR-206 expression was measured by RT-PCR. **B**–**D** The content of IL-1β, TNF-α, and IL-6 was evaluated by ELISA. **E**, **F** The NP cells apoptosis was detected by flow cytometry. **P < 0.01 versus control group, ##*P* < 0.01 versus LPS group, ^^*P* < 0.01 versus LPS + miR-206 mimics group

**Fig. 5:**
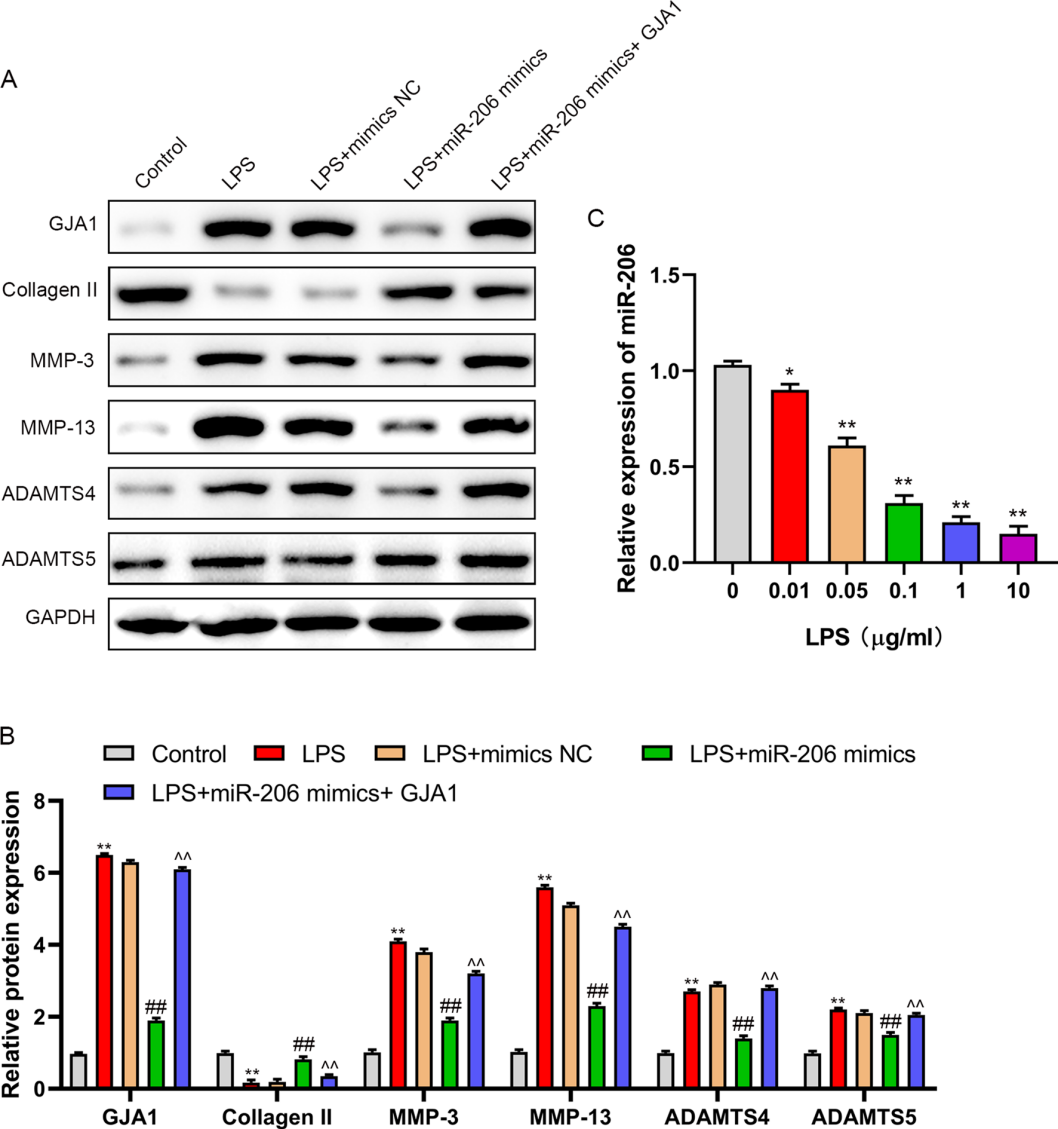
upregulation of miR-206 amplified ECM by targeting GJA1 in LPS-induced NP cells. **A**, **B** GJA1, Collagen II, MMP-3, MMP-13, ADAMTS4, and ADAMTS5 were evaluated by western blot. **C** miR-206 expression was measured by RT-PCR in NP cells induced by LPS (0, 0.01, 0.05, 0.1, 1, and 10 μg/ml). **P < 0.01 versus control group, ##*P* < 0.01 versus LPS group, ^^*P* < 0.01 versus LPS + miR-206 mimics group

In order to study the role of miR-206/GJA1 axis in NP cells, The cells were transfected with mimics NC, miR-206 mimics or miR-206 mimics + pcDNA-GJA1. Following that NP cells were treated for 24 h with LPS, and PBS treatment were used as control. The results showed that LPS-induced downregulation of miR-206 in NP cells, while which was reversed by transfection of miR-206 mimics. miR-206 mimics induced upregulation of miR-206 was attenuated by the co-transfection of pcDNA-GJA1 in LPS-induced NP cells (Fig. 4A). Transfection of miR-206 mimics reduced inflammatory factors (IL-1β, TNF-α and IL-6) (Fig. 4B–D), inhibited apoptosis (Fig. 4E, F), enhanced GJA1, type II collagen, decreased the expression of MMP-3, 13 and ADAMTS4 and 5 in LPS-induced NP cells, and the up-regulation of GJA1 weaken the effect of miR-206 mimics on LPS-induced NP cells (Fig. 5A, B). Moreover, the expression of miR-206 was decreased in LPS-treated NP cells (Fig. 5C). These results demonstrated that miR-206 inhibits LPS-induced NP cell injury by downregulating GJA1.

### MiR-206 improves IDD in vivo

**Fig. 6:**
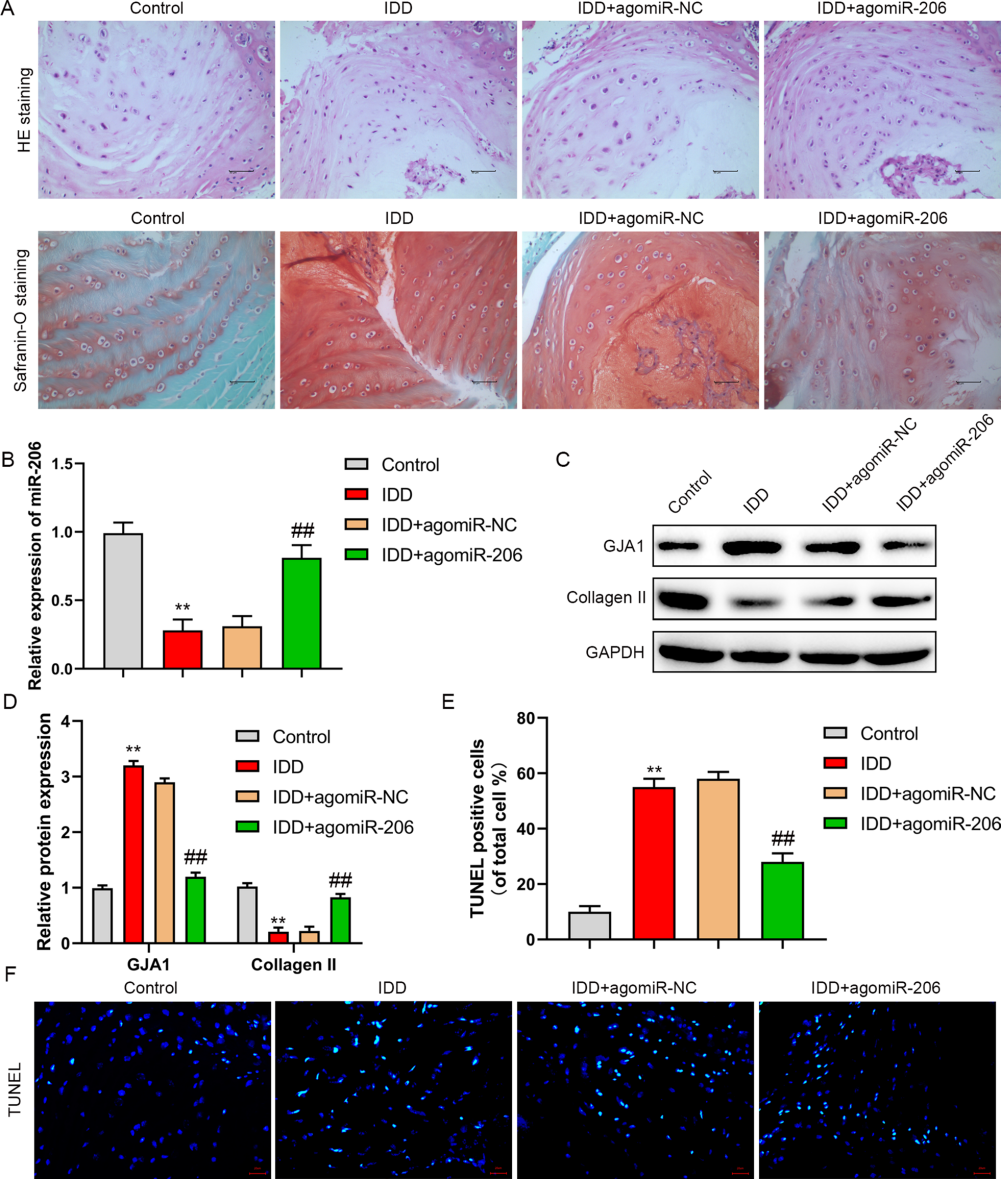
overexpression of miR-206 improved IDD in vivo. **A** Pathological changes were evaluated by HE staining and Safranin-O staining. **B** miR-206 expression was evaluated by RT-PCR. **C**, **D** GJA1 and Collagen II expression were detected by western blot. **E**, **F** The apoptosis was measured by TUNEL assay in IDD tissues. **P < 0.01 versus control group, ##*P* < 0.01 versus IDD group

IDD rat model was established by annulus fibrosus puncture. Administration of agomiR-206 could improve annulus fibrosus puncture induced IDD (Fig. 6A) and upregulate the expression of miR-206 in IDD tissues (Fig. 6B). Moreover, annulus fibrosus puncture induced upregulation of GJA1 expression and downregulation of type II collagen in IDD tissues, while agomir-206 reversed these effects (Fig. 6C, D). In addition, apoptosis in NP of IDD tissues was restrained by agomir-206 (Fig. 6E, F). These results showed that miR-206 overexpression could improve IDD in vivo.

## Discussion

Various biological agents, from matrix proteins and recombinant growth factors, to gene-based drugs, have been proposed in preclinical settings for the treatment of IDD [28, 29]. As housekeeping and regulatory noncoding RNAs, miRNAs and target genes have shown to be promising agents [21, 30]. Hence, miRNA-based therapies for IDD through targeting the key molecules might be the research trend in the future.

In this paper, GSE34095 dataset analysis and our study showed that GJA1 was highly expressed in IDD tissues compared with normal intervertebral disk tissues. Moreover, GJA1 expression was up-regulated in LPS-induced NP cells. In addition, GJA1 silencing could inhibit the expression of inflammatory factors and apoptosis, amplify the expression of type II collagen, reduce the expression of MMP-3 and 13, and increase the expression of ADAMTS4 and 5 in LPS-induced NP cells. Moreover, miR-206 targeting GJA1 was confirmed by luciferase reporter gene, and miR-206 was negatively correlated with GJA1 expression in IDD tissues. Furthermore, our results demonstrated that miR-206 inhibits inflammation, apoptosis and increases ECM by regulating GJA1 in IDD.

As one of the major proteins of connexin family, GJA1 played an important role in cardiovascular disease [31], and which was also reported to promote the metastasis of prostate cancer cells and breast cancer cells [32, 33]. Moreover, the high expression of GJA1 predicted a poor prognosis in colorectal cancer and was positively correlated with immune cell infiltration [34]. Besides, GJA1 knockout could provide neuroprotective effect in Aβ induced astrocytes, which indicated that GJA1 might improve Alzheimer's disease [35]. However, the function of GJA1 has rarely been studied in IDD. In our study, highly expressed GJA1 was found in IDD tissues and LPS-induced NP cells, and involved in promoting the secretion of inflammatory factors, inducing apoptosis and reducing ECM.

In recent years, the role of noncoding RNAs in musculoskeletal conditions has been concerned [36–38]. MiRNA can bind with target genes to inhibit the expression, and then affect the physiological function of cells [19, 39]. Hence, we explored the regulatory factors of GJA1 from the perspective of miRNA. Through targetscan, we found that the miRNAs targeting GJA1 included miR-1-3p, miR-206 and miR-613. Our results showed that only miR-206 is abnormally downregulated in IDD tissues, which is consistent with previous reports [40]. MiR-206, located on human chromosome 6, is a member of the muscle specific miR-1 family [41, 42]. MiR-206 was closely related to physiological processes such as cell proliferation, differentiation, apoptosis, invasion and metastasis [43–45]. MiR-206 could inhibit the proliferation and migration of lung adenocarcinoma cells by targeted binding to RMRP2 [46]. Moreover, BMSC-derived exosomes inhibit cell proliferation, migration and invasion by transmitting miR-206 to osteosarcoma cells [47]. MiR-206 targeted regulating the expression of connexin 43 (GJA1), thereby improving sepsis lung injury by reducing W/D ratio and BALF protein content [48]. In our study, the roles and relationships of miR-206 and GJA1 were explored in IDD. Our results similarly showed that miR-206 was downregulated in IDD tissues, miR-206 overexpression could improve LPS-induced NP cell injury in vitro, and upregulation of GJA1 could reverse the effect of miR-206 on LPS-induced NP cells, indicating that miR-206/GJA1 axis was involved in regulating progression of IDD. In vivo, agomiR-206 ameliorated the IDD induced by annulus fibrosus puncture, increased type II collagen, reducing apoptosis and up-regulated the expression of GJA1.

The etiology of IDD were involved in complex mechanisms. It was limited that we focused on miR-206/GJA1 in IDD. Hence, key molecules of multiple regulatory mechanisms need to be concerned. Moreover, highly specific miRNA in IDD should also be studied and applied in the future to improve the therapeutic effect. In addition, the intervertebral disks of rats are quite different from those of humans. Therefore, large animal models close to human size need to be studied in the future.

## Conclusion

Our data demonstrated that miR-206 targeting GJA1 contributes to the decrease of inflammatory factors, the inhibition of apoptosis, the increase of ECM in LPS-induced NP cells and annulus fibrosus puncture induced IDD. These studies provided a basis for miR-206/GJA1 to be a molecular marker or targeted index for the diagnosis and treatment of IDD.

## Data Availability

The datasets used and/or analyzed during the current study are available from the corresponding author on reasonable request.
